# Accessing social media: Help or hindrance for people with social anxiety?

**DOI:** 10.1177/2043808719837811

**Published:** 2019-04-04

**Authors:** Sophie E. Carruthers, Emma L. Warnock-Parkes, David M. Clark

**Affiliations:** University of Oxford, UK; King’s College London, UK; University of Oxford, UK; King’s College London, UK; University of Oxford, UK

**Keywords:** Facebook, negative thoughts, safety behaviors, social anxiety, social media, social networking

## Abstract

Despite increasing use of social media and the potential benefits for people with social anxiety (SA) disorder, little is known about the online experience of people with SA. Our study aimed to investigate the occurrence of cognitive and behavioral processes during a series of online and off-line Facebook (FB)-based tasks among individuals with high and low levels of SA. Sixty-one undergraduates with low or high SA were asked to use FB in a laboratory setting, to make an FB post, and to imagine three ambiguous FB scenarios. Participants with high SA reported higher anxiety throughout the study with an interaction effect, indicating greater relative increases in anxiety for those with high SA over low SA across tasks. The high SA group were more likely to negatively interpret the ambiguous FB scenarios than the low SA group. They also reported using more safety-seeking behaviors and having more negative thoughts. The findings suggest that the cognitive and behavioral processes that characterize socially anxious face-to-face interaction are also evident in online communication. Suggestions are made for the clinical implications of such findings.

## Introduction

A number of studies have indicated that people who experience high social anxiety (SA) are frequent users of social media and express a preference for technologically mediated communication (Caplan, 2007; Murphy & Tasker, 2011; Pierce, 2009). It has been hypothesized that the online social world may be less threatening and thus more attractive to people with SA disorder (SAD) (Caplan, 2007; Shepherd & Edelmann, 2005). Unlike a face-to-face interaction, in an online context, people can hide their expressions of anxiety (e.g., sweating, shaking, blushing) that they would typically be concerned about being noticeable, and more easily regulate the frequency, duration, and time lag of communication. This enables relationships to be built under more controlled conditions in a gradual way. Consequently, social media sites, such as Facebook (FB), could provide a platform for people with SA to establish a wider social network and build deeper relationships with others. However, this is only likely to happen if they actively interact with other people online, self-disclose, and do not engage with the negative thoughts and unhelpful behaviors that characterize, and interfere with, their social relationships in face-to-face communication (Clark & Wells, 1995; Rapee & Heimberg, 1997).

Research into cognitive–behavioral models of SAD (Clark & Wells, 1995; Rapee & Heimberg, 1997) demonstrates that during face-to-face social situations, individuals with SAD experience negative thoughts and images about how they think they are coming across (e.g., “I don’t have anything to say,” “I will say something stupid,” “I look anxious”). They also consider the catastrophic social consequences of their perceived poor performance (e.g., “I will be rejected”) (Hackmann, Surawy, & Clark, 1998; Stopa & Clark, 2000). During conversations, people with SAD remain predominantly self-focused, monitoring their performance rather than focusing on the social interaction (Mansell & Clark, 1999). Furthermore, individuals high in SA can engage in a range of self-protective strategies, referred to as safety behaviors, that are intended to prevent feared outcomes being realized. However, in fact, their use only prevents people from learning that the negative outcome they fear isn’t likely to occur. For example, someone who is concerned that other people will judge them as stupid might rehearse lots of “clever” things to say before a conversation. This prevents them from discovering that other people would find them perfectly interesting if they just said what came into their mind during a conversation. Instead, that individual believes the only reason they weren’t judged as stupid is because they had prepared topics in advance. In some cases, such rehearsed conversation can be experienced as awkward and off-putting to conversational partners. Empirical research with face-to-face interactions shows that safety behaviors play a critical role in maintaining SA (Wells et al., 1996), but little is known about their role in online communication.

If people with SAD were free of these processes in their online world, the Internet could provide a beneficial opportunity to build relationships while feeling more at ease. However, if interacting online also triggers the cognitive and behavioral processes that characterize and maintain face-to-face SA, the potential benefits of online communication will be lost unless we are able to provide guidance to help socially anxious individuals to use it in a more constructive way.

Questionnaire studies have provided preliminary evidence that the online interactions of individuals with SA are anxiety-provoking and tend to be characterized by problematic processes and strategies. McCord, Rodebaugh, and Levinson (2014) found that those with higher SA were more likely to report feeling anxious when interacting socially on FB, rather than passively using the site. Furthermore, Erwin, Turk, Heimberg, Fresco, and Hantula (2004) found that although participants reported greater ease with online communication, they also showed higher levels of passively observing Internet interactions, fear of negative evaluation of their Internet communications, and discomfort regarding being observed during Internet discussions. In addition, they found that existing negative beliefs (e.g., other people are critical or rejecting) were reinforced by online communication. A more recent study by Shaughnessy, Rocheleau, Kamalou, and Moscovitch (2017) found that people high in SA report more anxiety than those lower in SA when they anticipate interacting online and prefer to use methods of online communication that afford anonymity and a time lag between communicative reciprocity. Research also suggests that characteristics of online communication that allow control over self-presentation are likely to be among the most important for understanding how people higher in SA, and with more indicators of SA (e.g., fear of negative evaluation), use the Internet in a safety-seeking way (Kamalou, Shaughnessy, & Moscovitch, 2019). Finally, Ryan, Warnock-Parkes, and Clark (In publication) found that students with high SA reported experiencing more anxiety, more frequent negative cognitions (that they endorsed more strongly), interpreting ambiguous FB scenarios more negatively and using more safety behaviors on FB when asked in a questionnaire about their typical FB use. To the best of our knowledge, only one study has explored computer-based communication experimentally in this population. Markovitzky, Anholt, and Lipsitz (2012) focused on the effects of a brief Internet chat introduction on SA in a subsequent face-to-face contact; anxious arousal was reported by individuals with both low and high SA during the online chat.

This study aimed to extend these preliminary findings of questionnaire studies by using an experimental design in which high and low socially anxious individuals performed a series of online and off-line FB-based tasks in the laboratory. In line with predictions from cognitive–behavioral models of SA (Clark & Wells, 1995; Rapee & Heimberg, 1997) and research outlined above, it was hypothesized that when using FB, higher socially anxious individuals would (1) report greater anxiety levels, (2) report using more safety behaviors, (3) report a higher number of negative thoughts and (4) give more negative interpretations when asked to imagine hypothetical FB scenarios.

## Method

### Participants

Sixty-one University of Oxford students (31 female, 50.8%) meeting criteria for either high SA (*n* = 31, 50.8%) or low SA (*n* = 30, 49.2%) participated in the study. The categorization of high and low SA was determined by scores on the Brief Fear of Negative Evaluation Scale (BFNE). Following Garner, Mogg, and Bradley (2006), high SA participants were required to score 40 or above and low SA scored 30 or below. Thus, the BFNE was used to screen any individuals interested in participating; any individual scoring within the desired ranges were invited to participate, and those scoring 31–39 were informed they were not eligible for the study. Mean BFNE scores were 47.5 (*SD* = 4.9) and 26.5 (*SD* = 4.9) for the high and low SA groups, respectively. The mean age of participants was 20.10 years (range 18–25 years). Thirty-seven (60.7%) participants described themselves as White British, 11 (18.0%) as other White background, 4 (6.6%) as mixed White and Asian, 2 (3.3%) as other mixed background, 2 (3.3%) as Chinese, 1 (1.6%) as mixed White and Black Caribbean, 1 (1.6%) as Indian, 1 (1.6%) as Pakistani, 1 (1.6%) as African, and 1 (1.6%) chose not to disclose. The low SA group had 14 males and 16 females, and the high SA group had 16 males and 15 females. An independent sample *t*-test and χ^2^ tests confirmed there were no differences between the two groups in terms of age, gender, or ethnicity. As we wanted to capture typical FB behavior, we wanted to have participants who were familiar with using their FB account and so to take part in the study, they needed to access their FB account at least monthly. During screening, any participants who reported severe depression (20 or above on the Beck Depression Inventory (BDI) (Beck, Steer, & Brown, 1996) or suicidal ideation (scoring 2 or above on item 9 of the BDI) were excluded from the study but signposted to local services. Ethical approval was granted by the University of Oxford Central University Research Ethics Committee.

### Measures

#### The BFNE

A well-recognized 12-item measure of SA demonstrates both high internal consistency (*a* = .90–.91) and 4-week test–retest reliability (*r* = .75) in undergraduate samples (Leary, 1983). The BFNE was completed by potential participants as a screen of SA levels sent to them via a computer link that was e-mailed to them.

#### Beck Depression Inventory

BDI is a frequently used measure of depression symptomatology (Beck et al., 1996). We asked any potential participant to complete it in order to screen out anyone with high levels of depressive symptoms or suicidal ideation. Potential participants completed the BDI via a computer link that was e-mailed to them.

#### Self-rated anxiety

Participants were asked to report how anxious they felt at different points in the experiment using a 0–10 scale (0 = *not at all anxious*, 10 = *the most anxious I could feel*). To prevent a clear focus on anxiety, participants were asked about their anxiety along with nine other feelings (e.g., happiness, excitedness, self-consciousness). Only anxiety ratings were analyzed.

#### Facebook Safety Behaviours Checklist

To identify whether any safety behaviors were used during FB use in the laboratory, a checklist of 29 items was formed. Twenty of these items were taken from the preexisting Social Media Safety Behaviours Questionnaire (Warnock-Parkes & Clark, Unpublished-b). This questionnaire was developed by Warnock-Parkes and Clark following clinical interviews with patients with SAD about their social media use. It consists of 20 behaviors patients reported using when feeling anxious about their online interactions, such as rewording text multiple times, monitoring how others respond to what you post, and mentally storing up events or things to add to site. The Social Media Safety Behaviours questionnaire asks people to report how often they use these behaviors (never, sometimes, often, or always). However, the items here were presented in a checklist of whether they were used during the 10-min FB use, with each ticked item receiving a score of 1. Nine additional items were added by the current researchers who were relevant for FB use specifically, for example, “Censored photographs (e.g., by untagging or ‘hiding from timeline’).” These items were added as they seemed important potential safety behaviors that may be used while on FB that were not covered by the original Social Media Safety Behaviours Questionnaire. Following the 10-min FB task, the checklist was presented and participants were asked to tick those online safety behaviors that they used during the task. A total score was calculated representing the number of items ticked. The checklist is included in Supplementary Material. Internal consistency for the Facebook Safety Behaviours Checklist in this sample was *a* = 0.78.

#### Facebook Cognitions Checklist

A 29-item checklist measuring possible thoughts experienced during FB use (23 negative, 6 positive). All 29 items were taken from the Social Media Cognitions Questionnaire (Warnock-Parkes & Clark, Unpublished-a), which is a list of thoughts people may have when using social media. The original questionnaire asks people to report how commonly, on a 5-point scale, they experience particular thoughts and how much they believe them (0–100%). It consists of 23 negative items (e.g., “My life is boring compared to others,” “People will un-friend/follow me,” “Nobody will like what I add,” “People are watching/observing me,” “People think I am boring”) and 6 positive items (e.g., “People are interested in me”). The Social Media Cognitions Questionnaire was developed following clinical interviews with patients with SAD about their social media use. Six positive thoughts were included as a way of breaking up the negative thoughts to prevent autopilot responses. In this study, the items were presented as a checklist. Following the 10-min FB task, the checklist was presented, and participants were asked to tick any thoughts they had experienced during the task. Each thought endorsed by the participants scored 1 point and a total score was calculated. Only the 23 negative items were included in analyses. The checklist is included in Supplementary Material. Internal consistency for the Facebook Cognitions Checklist in this sample was *a* = 0.74.

#### Use of FB questionnaire

A brief questionnaire was devised by the researchers to assess a range of factors including the frequency of FB usage (daily, every few days, weekly, monthly), the amount of time spent on FB each day (less than 30 min, up to an hour, 1–2 hr, 2–3 hr, more than 3 hr), number of online friends, whether participants monitored the number of online friends, and the proportion of friends the participant was conscious of viewing their posts. Participants were also asked whether they preferred to communicate face-to-face or via FB with close friends, close family, extended family, or acquaintances.

#### FB Scenarios Questionnaire

The Facebook Scenarios Questionnaire followed a format (Clark et al., 1997) that has frequently been used in psychopathology research to assess interpretations. It consisted of three ambiguous hypothetical FB scenarios each with a potential risk of negative interpretation. The three scenarios were “Somebody leaves a jokey comment on one of your entries on FB,” “You post something to Facebook (e.g., a status update or a profile picture) and at the end of the day you see that nobody has ‘liked’ or commented on it,” and “You discover someone has deleted you as a friend on Facebook.” Participants were asked to have their FB profile open on their screen and to imagine each scenario happening to them. They were then asked to rate their anxiety on a scale ranging from 0 = *not at all anxious* to 10 = *the most anxious I could feel*. Participants then turned to the next page of the questionnaire where they were asked to rank order the likelihood that three alternative explanations would come to mind: one alternative was always negative and two were neutral. Participants were then asked to rate the extent to which they would believe each of the explanations, on a 1–8 (1 = *don’t believe it at all*, 8 = *completely believe it*) scale. For analysis, a score of 1, 2, or 3 was given depending on whether the negative explanation ranked first, second or third, respectively. The order and belief ratings and anxiety ratings were averaged across all three scenarios.

### Procedure

Interested potential participants were e-mailed the two screening questionnaires and an information sheet about the study. Any eligible participant was invited to come into the laboratory for the study. Informed consent was obtained for all participants prior to the study. On arrival in the laboratory, participants were given a booklet of instructions and questions. The experimenter kept interaction with the participants to a minimum and advised the participants to follow the instructions written in the booklet and to ask whether they had any questions. The experimenter remained in the room but sat the other side of a screen so the experimenter and participant could not see each other, nor the experimenter see the computer screen. First, participants were instructed to complete the use of FB questionnaire, followed by a baseline anxiety rating. Participants were then instructed to complete the first task which was to use FB for 10 min as they would typically on a laboratory computer. The participant was asked to inform the experimenter when they started this section so that it could be timed. The experimenter then informed the participant when 10 min was over and the participant continued to follow the instructions in the booklet. Following this first task, participants were asked to consider their 10 min of FB use and to rate their anxiety accordingly. Then they were asked to indicate whether they experienced the Facebook Safety Behaviours Checklist and Facebook Cognitions Checklist. For the second task, participants were asked to make an FB post (with suggestions given to post on someone’s wall, make a status update or share a link) and to re-rate their anxiety following this task. Finally, for the third task, participants considered a series of hypothetical FB-related scenarios by completing the Facebook Scenarios Questionnaire and were instructed to re-rate their anxiety when considering each scenario. Following the completion of the study, all participants received a debriefing document along with £5 payment for their travel costs.

### Statistical analyses

Analyses were conducted using Stata (version 14). Anxiety ratings following each experimental task were analyzed using random intercept linear models (i.e., multilevel regression models), with participants as random effects and experimental phase and group as fixed effects. Models were estimated using the *mixed* command with maximum likelihood estimation. Statistical significance of the interaction term was tested with a likelihood ratio test. To explore the best model fit, two models were compared with and without the interaction term against χ^2^ with one degree of freedom. We tested main effects of group (low SA vs. high SA), experimental phase (baseline, 10-min typical FB use, FB entry, FB scenarios), and group-by-experimental phase interaction. Significant effects were followed up with post hoc contrasts for between-group differences in anxiety which accounted for multiple comparisons with Bonferroni corrections. Ratings of anxiety during FB and face-to-face interaction on the Use of FB questionnaire were analyzed using a mixed analysis of variance (ANOVA) comparing between-group (high vs. low SA) and within-group (face-to-face vs. FB) ratings. For analyses of questionnaire data, χ^2^ tests and independent *t*-tests were used to compare group differences. For *t*-tests, effect size was calculated using Cohen’s *d* (where 0.20 = small, 0.40 = moderate, 0.80 = large; Cohen, 1988). There was a small amount of missing data at an item level with a maximum of four pieces of missing data (anxiety rating following making a FB entry). All missing data points were due to missed items on the questionnaires. Due to the small amount of missing data, no imputation was conducted. The anxiety ratings analysis included all observed data according to the maximum likelihood estimation. *T*-test analyses excluded missing data points. *N* values are stated for each analysis.

## Results

Mean, *SD*s, and effect sizes are reported in Tables 1 and 3 or in the text below.

**Table 1.** Mean anxiety scores by experimental phase (baseline, 10-min general FB use, making an FB post and FB scenarios) for both high and low SA groups.

Experimental phases	High SA	Low SA	Cohen’s *d*
	*M* (*SD*)	*M* (*SD*)	
	*(n)*	*(n)*	
Baseline	2.57 (2.01)	1.37 (0.85)	0.78
	(*n* = 30)	(*n* = 30)	
Ten-minute FB use	2.94 (1.81)	1.31 (0.81)	1.16
	(*n* = 31)	(*n* = 29)	
FB post	4.27 (2.33)	2.08 (1.50)	1.12
	(*n* = 30)	(*n* = 26)	
FB scenarios	4.73 (2.08)	2.13 (1.27)	1.51
	(*n* = 31)	(*n* = 29)	

*Note*. M = mean, *n* = number of individuals, SD = standard deviation, FB = Facebook, SA = social anxiety.

### Self-reported use of FB

The majority of participants reported using FB daily (98%, *n* = 60), more than 4 times a day (60%, *n* = 36), for up to 10 min at a time (75%, *n* = 46) and for up to an hour a day in total (69%, *n* = 42). Nearly a third of participants (28%, *n* = 17) reported being on FB for more than an hour a day. The χ^2^ analyses revealed that there were no differences between high and low SA individuals in the frequency of visits to FB per day (*p* = .148) or in the total amount of time spent on FB per day (*p* = .485).

Participants reported that they had a mean number of 644 friends (*SD* = 315.15). Independent *t*-tests were used to compare the number of friends for both high (*M* = 653.39, *SD* = 378.31) and low SA (*M* = 633.83, *SD* = 239.22), and no differences were found between the two groups (*p* > .05, *d* = 0.06). However, when participants were asked whether they frequently monitor the number of friends that they have on FB, nine (29%) high SA participants reported they did, compared to two (3.7%) low SA participants.

All participants reported that they would prefer to communicate off-line (rather than through FB) with close friends, but 20 (64.5%) high SA and 12 (40%) low SA participants reported that they would prefer to use FB to communicate with people they knew less well (acquaintances). When a χ^2^ test was performed, this difference was significant, χ^2^(1) = 4.98, *p* = .026.

The high SA group reported comparing themselves to others on more domains (including number of friends, number of photos, type of photos, events, how much fun is being had, number of likes on posts, number of comments on posts and amount of personal information shared) than the low SA group (high SA: *M* = 3.74, *SD* = 1.97; low SA: *M* = 1.33, *SD* = 1.56; *t*(59) = −5.29, *p* < .001, *d* = 1.36). Furthermore, when asked what proportion of their friends they felt were observing their posts, the high SA group reported feeling conscious of a higher percentage of their friends seeing their posts (*M* = 17.60, *SD* = 25.01) than the low SA group (*M* = 2.59, *SD* = 4.21). A *t*-test revealed that this difference was significant with equal variances not assumed, *t*(56) = −3.13, *p* = .003, *d* = 0.83).

### Anxiety when using FB

Before completing the laboratory FB tasks, participants were asked in the use of FB questionnaire to rate their general levels of anxiety when interacting face-to-face with other people and then on FB. People with low SA reported a mean anxiety score of 2.20 (*SD* = 1.13) in face-to-face situations and 1.93 (*SD* = 1.30) when using FB. People with high SA reported a mean anxiety score of 3.77 (*SD* = 1.78) in face-to-face situations and 3.48 (1.91) on FB. A mixed ANOVA comparing between-group (high vs. low SA) and within-group (face-to-face vs. FB) anxiety ratings found a significant main effect of group (*p* < .001), no main effect of mode of communication (face-to-face vs. FB; *p* = 0.221), and no interaction effect (*p* = 0.958), *F*(62) = 2.90, *p* < .001. This indicates the high SA group reported higher anxiety than the low SA group across face-to-face and FB communication. Ratings of anxiety between FB and face-to-face communication did not differ for either group.

Anxiety was also rated during the different phases of the experimental FB task (see Table 1). To explore these data, two models were developed: Model 1 without an interaction term and Model 2 with an interaction term between experimental task and group. The likelihood ratio test (Model 2 compared with Model 1) confirmed that the interaction term significantly improved the model fit, *p* = 0.015, so Model 2 results are subsequently reported (Table 2).

**Table 2.** Fixed effects of group, experimental phase and the interaction between group and experimental phase on anxiety self-ratings of participants.

	Model including interaction term		
	Estimate	*SE*	*p*
Intercept	1.37	0.30	<.001
Main effect: group			
Low SA	Reference		
High SA	1.16	0.43	0.006
Main effect: experimental phase			
Baseline	Reference		
Ten-minute FB use	−0.001	0.32	0.997
FB entry	0.78	0.34	0.021
FB scenarios	0.81	0.32	0.012
Interactions			
High SA × 10-min FB Use	0.41	0.45	0.368
High SA × FB Entry	0.98	0.46	0.036
High SA × FB Scenarios	1.39	0.45	0.002

*Notes*. Estimate refers to the coefficient estimate. Low SA and baseline anxiety were reference groups. High SA represents the difference between the low SA and high SA groups. Ten minutes represent the difference between baseline and 10 min of typical FB use, FB entry represents the difference between baseline and after participants had made an online FB post and FB scenarios represents the difference between baseline and after participants have completed the three hypothetical FB scenarios. The interactions follow the same principle, that is, high SA × 10-min FB represents the interaction between group and the difference between baseline and 10 min of FB use. Contrast includes Bonferroni corrections. FB = Facebook, *p* = significance level, SA = social anxiety, *SE* = standard error.

**Figure 1.**
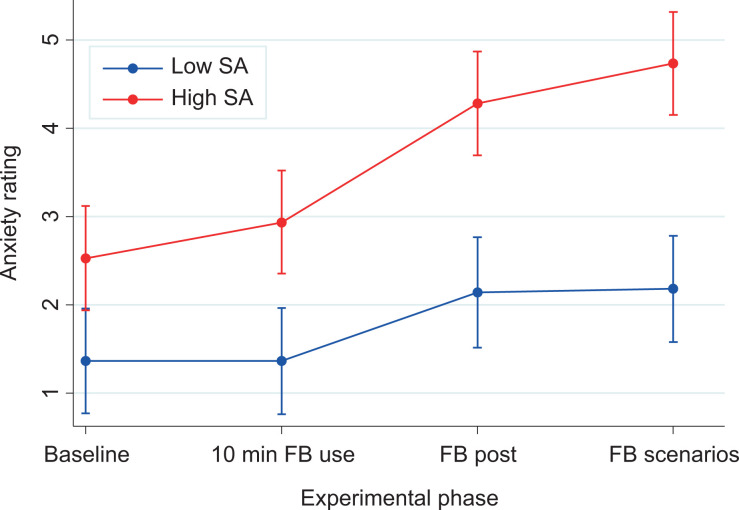
Anxiety ratings of participants from the low (blue) and high (red) SA groups across the four experimental phases with 95% confidence intervals. Anxiety ratings were made on a 0–10 scale (0 = *not at all anxious*, 10 = *the most anxious I could feel*). FB = Facebook, SA = social anxiety.

There were significant main effects of group, χ^2^(1) = 32.57, *p* < .001, and of experimental phase, χ^2^(3) = 65.25, *p* < .001, and a significant interaction effect, χ^2^(3) = 10.85, *p* = .013, between the group and the experimental phase (Figure 1).

Post hoc contrasts confirmed that the high SA group reported significantly higher anxiety ratings than the low SA group at all four experimental phases (Table 2). This result therefore supports our hypothesis that individuals with high SA experience greater anxiety when using FB than individuals with low SA. In addition, though both the low SA (*p* = .009) and high SA (*p* < .001) groups significantly increased in anxiety over the course of the experimental phases (Table 2), the significant interaction term suggests the increase in anxiety was differential between the two groups. Visual inspection of the interaction plot (Figure 1) reveals there was a higher relative increase in anxiety over the course of the experimental phases for the high SA group compared to the low SA group. A post hoc contrast demonstrated a larger overall increase in reported anxiety from baseline to the final task (scenarios) for the high SA group than the low SA group (*p* = 0.007). Individuals with high SA, therefore, reported significantly higher anxiety throughout the tasks and experienced a greater relative increase in anxiety over the course of the tasks. Although this needs to be interpreted with caution, this may also support our hypothesis that individuals with high SA experience greater anxiety when using FB than individuals with low SA.

### Safety behaviors using FB

To explore differences in the reported number of safety behaviors used during the 10-min of FB use between the low and high SA groups, an independent-samples *t*-test was used (see Table 3 for mean values). The reported number of safety behaviors in the 10-min FB use was higher among the high SA group than the low SA group, *t*(59) = −4.32, *p* < .001, *d* = 1.11. These findings support our hypothesis that individuals with high SA would experience a higher number of safety behaviors than the low SA group.

**Table 3.** Safety behaviors and negative thoughts reported following 10-min general FB use in the laboratory.

	High SA	Low SA		
Variable	*M* (*SD*)	*M* (*SD*)	*t*	Cohen’s *d*
	*(n)*	*(n)*		
Safety behaviors	4.97 (2.52)	2.23 (2.42)	−4.32***	1.11
	(*n* = 31)	(*n* = 30)		
Negative cognitions	3.13 (2.80)	0.80 (1.06)	−4.33***	1.09
	(*n* = 31)	(*n* = 30)		

*Note. d* = Cohen’s *d*, M = mean, *n* = number of individuals, *p* = level of significance, *SD* = standard deviation, *t* = *t*-test statistic, FB = Facebook, SA = social anxiety.

*p < .05; **p < .01; ***p < .001.

### Negative thoughts using FB

To determine whether there were differences in the reported number of negative thoughts after the 10-min of FB use between the low and high SA groups, an independent-samples *t*-test was run (see Table 3 for mean values). With equal variance not assumed, the reported number of negative thoughts in the 10-min FB use was higher among the high SA group than the low SA group, *t*(38.78) = −4.33, *p* < .001, *d* = 1.09. These findings support our hypothesis that individuals with high SA would experience a higher number of negative thoughts than the low SA group.

### Negative interpretations of FB scenarios

Ranking ratings for the FB scenarios indicated that high SA individuals (*M* = 1.82, *SD* = .45, *n* = 31) were more likely to make negative interpretations than the low SA group (*M* = 2.38, *SD* = .36, *n* = 29), *t*(58) = 5.27, *p* < .001, *d* = 1.38. The high SA group also gave higher belief ratings for the negative interpretations than the low SA group (high SA: *M* = 4.87, *SD* = 1.10, *n* = 31; low SA: *M* = 3.76, *SD* = 1.12, *n* = 29), *t*(58) = −3.87, *p* < .001, *d* = 1.00. These findings of large effect support our hypothesis that people with high SA would be more likely to jump to negative interpretations when asked to imagine hypothetical FB scenarios with a risk of negative evaluation.

## Discussion

Our study aimed to be the first of its kind to use experimental methods and questionnaires to investigate the cognitive and behavioral processes experienced by individuals with high and low SA, including during live use of FB. We found that, during a laboratory FB task, individuals with higher SA tended to think and behave in a similar fashion to the way in which they would approach normal face-to-face interactions. In particular, our results suggest that during FB use individuals with high SA experience greater levels of anxiety, conduct more safety behaviors, have more negative thoughts, and are more likely to negatively interpret ambiguous scenarios than individuals with low SA. Participants with high SA also self-reported engaging with FB for similar amounts of times and having equal numbers of friends to those with low SA. Therefore, despite experiencing greater levels of anxiety, individuals with high SA continue to interact with FB as often and with as many friends as those low in SA. Our findings contribute to the research on SA online by suggesting that a similar profile of cognitive–behavioral processes to those considered to maintain SA off-line also affects online communication, even while individuals with high SA engage in the use of social media.

Across the series of laboratory FB tasks, high SA participants experienced more anxiety and a greater relative increase in anxiety over the course of the study. Considering the significant interaction effect, visual inspection of the data suggests a greater increase in anxiety for the high SA group relative to the low SA group at the time of the task that asked participants to post an FB entry. If this is the case, such a finding is in line with research suggesting anxiety is particularly high for those with SA at times of heighted risk of negative evaluation (Clark & Wells, 1995; Erwin, Turk, Heimberg, Fresco, & Hantula, 2004), and when socially interacting on FB as opposed to during passive use (McCord, Rodebaugh, & Levinson, 2014). It is important to consider that the sequential design of the tasks may mean that the previous tasks may have an influence over the anxiety rating provided at the time of the FB task; however, it should be noted that this does not explain the difference observed between the two groups. The interaction with an experimenter (though kept to a minimum) as a potential confound should also be considered.

The high SA group also used more safety behaviors, experienced more negative thoughts, and were more likely to interpret ambiguous FB scenarios negatively. These findings are consistent with studies that have started to explore the presence of maladaptive cognitive and behavioral patterns for people with higher SA while using social media (Erwin et al., 2004; Ryan, Warnock-Parkes, & Clark, In Publication) and support the application of the cognitive model of SAD (Clark & Wells, 1995) for online interactions. Taken together with our finding that self-reported general levels of anxiety did not differ between within participants’ online and off-line interactions, the results indicate that socializing on FB may not be the safe haven some propose for people with SA and can at times be just as anxiety-provoking and trigger the same cognitive and behavioral processes as face-to-face interactions.

Despite literature suggesting that social media may be more attractive to people with SAD (Caplan, 2007; Shepherd & Edelmann, 2005) and findings reporting those with high SA tend to express a preference for using and spending more time on social media (Murphy & Tasker, 2011; Pierce, 2009), our high SA group did not report using FB more frequently than those with low SA. This is not the first study to identify that levels of SA do not necessarily relate to time using the FB site. Fernandez, Levinson, and Rodebaugh (2012) found that SA was not correlated with self-reported time on FB. It may be that although those high in SA engage with FB as frequently and with similar numbers of friends, their style of use may be more passive in order to avoid the increases in anxiety related to more active social interaction as seen in our study following the FB post. This would be in line with previous findings suggesting a preference for passive use that affords anonymity (Erwin et al., 2004; McCord et al., 2014; Shaughnessy, Rocheleau, Kamalou, & Moscovitch, 2017).

In contrast to other studies, our high SA group did not express a general preference for using social media. However, high SA participants reported a greater preference (64.5% compared to 40.0% of the low SA group) for communicating with acquaintances via FB than face-to-face than those low in SA. If FB seems more appealing when interacting with less familiar people only, this might explain discrepant findings with previous studies that have asked participants more generally about whether they prefer to interact online or offline (Caplan, 2007; Murphy & Tasker, 2011; Pierce, 2009).

### Clinical implications

By engaging in the same unhelpful cognitive and behavioral processes online that maintain SA face-to-face (such as limiting posts or overly preparing what to say), people with SA may inadvertently limit the potential advantages that the Internet can offer them to build deeper relationships in a gradual and controllable way. People experiencing high SA might benefit further from their online interactions if they actively use the site to increasingly share more and interact with others rather than passively observing, while not overly focusing on or monitoring their own posts. Instead, it might be important to write more spontaneously without excessive censoring and use of other safety behaviors. As in face-to-face social relationships, we get to know other people more deeply and feel more accepted when we share more of ourselves. SA online is likely to persist unless people can start to change the way that they use social media. Cognitive therapy SAD is a leading, NICE recommended treatment for SAD focused on face-to-face interactions (National Institute for Health and Care Excellence, 2013) that explicitly encourages people to drop these strategies. That these strategies are also active in online interactions is an important discovery and clinicians should also pay attention to a patient’s online social environment in treatment in order to maximize clinical improvement.

Those with higher levels of SA in the general public may also be helped by an awareness of the effect of some of these unhelpful thinking styles and safety behaviors and to experiment with dropping them. It might be beneficial for online SA support organizations to provide information (e.g., through use of blogs or online articles) about how people could experiment with dropping unhelpful strategies, in order to have a more fulfilling experience of social media.

### Limitations and future research

It is important to consider these findings in the context of a number of limitations. Firstly, as this is a student sample, generalizing findings to a clinical population is somewhat limited and further experimental research into the use of social media within a diagnosed SA sample would be of benefit. Although we have included key checklists in Supplementary Material, a number of the measures we have used were novel for this study and in some cases are adapted from currently unpublished tools limiting the available validation. Furthermore, the reliance on self-report for all information in the study is a weakness of the design. Future studies may seek to explore validating self-report of FB use by asking participants to show them the information on their FB account. Given increasing research into physiological correlates of anxiety and use of smartwatches, future research incorporating such a measure (e.g., skin conductance or heart rate) would be informative. Finally, we focused solely on FB use and not on other forms of social media. Subsequent studies should consider a broader range of forums and sites that afford different opportunities and styles of interaction. Although the current study informs on the cognitive and behavioral processes involved in social media use, the extent of passive (e.g., scrolling through the time line, looking at friends’ pages) versus more active social interaction (e.g., updating the status, posting an entry on friend’s time line) has not been explored experimentally and would benefit from further investigation.

## Conclusions

Social media is increasingly becoming a day-to-day part of our social world. However, relatively little is known about how people with high SA experience online interaction. In the first study of its kind, we have found using an experimental task and live use of FB that the cognitive and behavioral processes that maintain face-to-face SA are also present in online interaction: the online world may not be the safe haven some propose for people with SA. If people with high SA could be alerted to the problematic ways in which they may think and behave on social media, it is possible they could change the way they engage online in order to build wider social networks and deeper relationships with others.

## Supplemental material

Supplementary_materials_(1) - Accessing social media: Help or hindrance for people with social anxiety? Supplementary_materials_(1) for Accessing social media: Help or hindrance for people with social anxiety? by Sophie E. Carruthers, Emma L. Warnock-Parkes, and David M. Clark in Journal of Experimental Psychopathology
