# Supplementary material for: Accessing social media: Help or hindrance for people with social anxiety?
Source: J Exp Psychopathol. 2019 Apr 4;10(2):2043808719837811. doi: 10.1177/2043808719837811 (PMC7086304; doi:10.1177/2043808719837811)
Supplement: Supplementary_materials_(1) - Accessing social media: Help or hindrance for people with social anxiety? [file Supplementary_materials_(1).pdf]

Supplementary materials

Facebook Safety Behaviours Checklist

Please tick if this behaviour has occurred in the past 10 minutes of Facebook use

Repeatedly edited or retyped what you posted	
Carefully selected the words or content you added	
Made an effort to get what you said right	
Planned what to put on the site in advance	
Mentally stored something to add to Facebook at a later point	
Exaggerated something to appear better to others	
Increased your privacy settings	
Censored photographs (e.g. by untagging or ‘hiding from timeline’)	
Avoided posting a status update	
Compared your profile to others	
Tried not to attract attention	
Checked back over posts you added	
Made an effort to come across well	
Avoided adding anything to Facebook	
Tried to picture how you appear to others	
Tried not to add anything too personal	
Avoided going online to Facebook chat	
Checked that you were coming across well	
Tried to act normal	
Checked words or information you had used were accurate (e.g. Google or spell check)	
Used interesting photographs taken purposefully for Facebook	
Limited information about your likes and dislikes	
Went over something you had posted on Facebook in your mind afterwards	
Censored what you added to Facebook	
Made a special effort to appear witty, intelligent or interesting	
Monitored how others responded to your posts	
Thought of something to post or comment but deciding not to do so	
Turned off Facebook chat to all or some friends	
Filtered who could see some posts	

<u>Facebook Cognitions Checklist</u>

Please tick if the thought has crossed your mind in the past 10 minutes of Facebook use

I am unlikeable	
People can tell I am nervous	
I am foolish	
People will reject me	
I am inadequate	
I am equal to others	
I don't have as many friends as others	
I am unable to write properly	
People are not interested in me	
I'm coming across badly	
I will make a mistake	
I come across well	
People won't like me	
People are interested in me	
I am vulnerable	
People on Facebook are watching me	
I am weird or different	
People will judge me negatively	
I am acceptable to others	
When compared to other people I come off worse	
People think I am boring	
I write inappropriate things	
I write stupid things	
People enjoy what I add on Facebook	
My life is boring compared to others	
People like what I put on Facebook	
No one will like what I do/add	
People know I have been looking at their profile	
People will unfriend me	
Other:	
